# Xanthone Biosynthetic Pathway in Plants: A Review

**DOI:** 10.3389/fpls.2022.809497

**Published:** 2022-04-08

**Authors:** Juwairiah Remali, Idin Sahidin, Wan Mohd Aizat

**Affiliations:** ^1^Institute of Systems Biology (INBIOSIS), Universiti Kebangsaan Malaysia, Bangi, Malaysia; ^2^Faculty of Pharmacy, Universitas Halu Oleo, Kendari, Indonesia

**Keywords:** synthesis, phytochemical, phenolic, flavonoid, biosynthesis, 9H-xanthen-9-one

## Abstract

Xanthones are secondary metabolites rich in structural diversity and possess a broad array of pharmacological properties, such as antitumor, antidiabetic, and anti-microbes. These aromatic compounds are found in higher plants, such as Clusiaceae, Hypericaceae, and Gentianaceae, yet their biosynthetic pathways have not been comprehensively updated especially within the last decade (up to 2021). In this review, plant xanthone biosynthesis is detailed to illuminate their intricacies and differences between species. The pathway initially involves the shikimate pathway, either through L-phenylalanine-dependent or -independent pathway, that later forms an intermediate benzophenone, 2,3′,4,6-tetrahydoxybenzophenone. This is followed by a regioselective intramolecular mediated oxidative coupling to form xanthone ring compounds, 1,3,5-trihydroxyxanthone (1,3,5-THX) or 1,3,7-THX, the core precursors for xanthones in most plants. Recent evidence has shed some lights onto the enzymes and reactions involved in this xanthone pathway. In particular, several biosynthetic enzymes have been characterized at both biochemical and molecular levels from various organisms including *Hypericum* spp., *Centaurium erythraea* and *Garcinia mangostana.* Proposed pathways for a plethora of other downstream xanthone derivatives including swertianolin and gambogic acid (derived from 1,3,5-THX) as well as gentisin, hyperixanthone A, α-mangostin, and mangiferin (derived from 1,3,7-THX) have also been thoroughly covered. This review reports one of the most complete xanthone pathways in plants. In the future, the information collected here will be a valuable resource for a more directed molecular works in xanthone-producing plants as well as in synthetic biology application.

## Introduction

**FIGURE 1:**
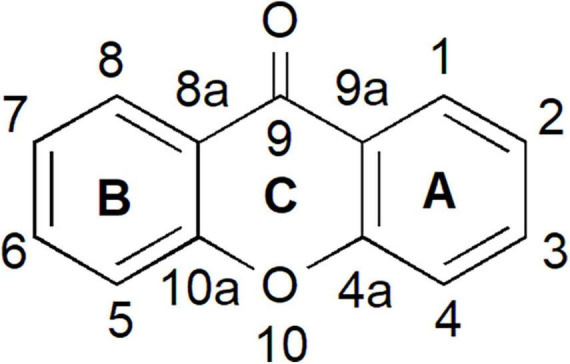
Xanthone is a phenolic compound with a planar dibenzo-γ-pyron scaffold. It contains conjugated aromatic ring system that is composed of two rings; carbons 1–4 (ring A) and carbons 5–8 (ring B) and attached together through an oxygen atom and a carbonyl group (ring C) to form the basic skeleton of xanthone known as 9H-xanthen-9-one.

Xanthones have been studied for more than five decades and are known to possess diverse structures, functions, and biochemical activities (Carpenter et al., 1969; Sultanbawa, 1980; Bennett and Lee, 1989; Peres et al., 2000; El-Seedi et al., 2010). The word “xanthone” originated from the Greek word “xanthos,” meaning yellow. Xanthones are a class of plant phenolic compound with C6-C1-C6 carbon skeletal structure (Figure 1). The two aromatic rings in the xanthone basic skeleton are numbered and designated based on their biosynthetic origins in higher plants. A-ring is acetate-derived and its carbons are numbered 1–4 whereas B-ring is derived from shikimate pathway and the carbons are numbered 5–8 (Ramawat and Mérillon, 2013; Wezeman et al., 2015; Pinto et al., 2021). Both of these rings can fuse together through an oxygen atom and a carbonyl group to form the simplest class of xanthone known as 9H-xanthen-9-one that is also symmetric with the skeleton of dibenzo-γ-pyron (Figure 1; El-Seedi et al., 2009, 2010; Wezeman et al., 2015).

Xanthones from natural sources contain various substituents on these two benzene rings, thus leading to wide structural diversity with a broad spectrum of activities (Sousa and Pinto, 2005; Tovilovic-Kovacevic et al., 2020). They are mainly classified into six main categories, namely, simple xanthones, glycosylated xanthones, prenylated xanthones, xanthonolignoids, bis-xanthones, and miscellaneous xanthones (Vieira and Kijjoa, 2005; El-Seedi et al., 2009). However, plants mostly produce xanthones in the first three categories (El-Seedi et al., 2009). Xanthones are also organized based on their quantity of oxygenation derivatives, prenylation, and glycosylation patterns. For instance, the simple oxygenated xanthones are further subdivided based on the level of oxygenation, such as non-, mono-, di-, tri-, tetra-, penta-, and hexa-oxygenated substances (Velíšek et al., 2008; El-Seedi et al., 2010; Masters and Bräse, 2012). Such a variety of xanthone structures allow their broad array of valuable pharmacological activities including as anti-microbes, antioxidant, anti-inflammation, antitumor, antidiabetic, anti-arthritis, as well as gastro-, liver- and cardio-protectant (Aizat et al., 2019; Marzaimi and Aizat, 2019).

Although xanthone structures and chromatographic patterns are comparable to that of flavonoids, xanthones are present in only a few restricted species (Jensen and Schripsema, 2002; Vieira and Kijjoa, 2005). For example, xanthones can only be isolated from 20 families of higher plants of which most of them reside within Clusiaceae, Hypericaceae, and Gentianaceae families (Tovilovic-Kovacevic et al., 2020), and to some extent in Calophyllaceae family (Gómez-Verjan et al., 2017; Zailan et al., 2021). Prenylated xanthones can be mainly isolated from different *Garcinia* species (Clusiaceae). These include α-mangostin, 7-*O*-methylgarcinone E, gartanin, garcinone E, and tovophyllin A from *Garcinia mangostana* (mangosteen; Ying et al., 2017; Mamat et al., 2020) and oblongifolixanthone A from *Garcinia oblongifolia* (also known as Lingnan Garcinia; Shan et al., 2012; Khan et al., 2020). *Hypericum* spp., such as *Hypercium calycinum* and *Hypercium sampsonii* from the Hypericaceae family, also produce prenylated xanthones, such as hyperxanthone E and patulone (Fiesel et al., 2015; Nagia et al., 2019). These prenyl groups can contribute to increasing bioactivities of xanthone due to their increasing lipophilicity to interact with biological membranes (Chen et al., 2017). Pendant sugars are also found as a structural feature of dimers in xanthones, for example, puniceaside C (xanthone glycosides from *Swertia punicea*; Zafar and Wang, 2018) and tocotrienol quinone dimer isolated from *Garcinia nigrolineata* (Raksat et al., 2019). In addition to that, C-glycosylated xanthones, such as mangiferin and isomangiferin, can be isolated from *Mangifera indica* (mango; Haynes and Taylor, 1966) and the aerial parts of *Anemarrhena asphodeloides* (Chinese herbs; Aritomi and Kawasaki, 1970). Mangiferin is also shown to be present widely among ferns (lower plants), such as *Polypodiopsida* or *Polypodiophyta* (Bennett and Lee, 1988). The different xanthone types from plants and their uses are described recently by Tovilovic-Kovacevic et al. (2020), and readers are directed to their review for more details.

Other than plants, xanthones can also originate from fungi, such as *Aspergillus*, *Helminthosporium*, *Penicillium*, and *Pyrenochaeta* (Bräse et al., 2009; Schätzle et al., 2012; Yoiprommarat et al., 2020), as well as lichens, such as *Parmelia* (Tuong et al., 2019), and bacteria, such as *Streptomyces* (Masters and Bräse, 2012). Interestingly, several simple methylated xanthones (1-methylxanthone, 2-methylxanthone, 3-methylxanthone, and 4-methylxanthone) can also be found from fossil fuels (Oldenburg et al., 2002; Masters and Bräse, 2012). The number of xanthone compounds from natural products has risen by 100 times over the last decades (El-Seedi et al., 2009). By July 2020, the number of xanthones recorded in the Dictionary of Natural Product has reached a staggering 2221 compounds^1^ (Supplementary Table 1), showing the diversity of this compound class. Nevertheless, the source of xanthone from plants remains dominant that counts approximately 80% of total natural xanthones in contrast to non-lichenized fungi (15%) and lichens (5%; Le Pogam and Boustie, 2016), and hence they become interesting subjects for xanthone studies.

Previous xanthone investigation encompassed various studies including structure–activity relationships (Pinto et al., 2005, 2021), xanthone production using biotechnological approaches (Gaid et al., 2019), *in vitro* and *in vivo* biological evaluation (Ovalle-Magallanes et al., 2017; Aizat et al., 2019), as well as structural (Wu et al., 2009) and isolation (Wang et al., 2013) studies. Given the increasing global demand for medicinal compounds, it is important to understand the biosynthesis of specialized metabolites, such as xanthones, as complete as possible, so that appropriate plant chemical resources can be established or developed in the future (Rai et al., 2017; Jamil et al., 2020). Thus, in parallel of their intriguing structural, biochemical, and pharmacological properties that they possess, this review aims to focus on gathering and updating the xanthone biosynthetic pathway in plants. This review also highlights the identification of enzymes involved in the pathway and describes their possible arrangement. In the future, this knowledge may improve synthetic biology efforts for a more sustainable production of plant natural products, such as xanthones or their benzophenone precursors, through metabolic engineering.

Several previous plant xanthone reviews have detailed various research aspects, for instance in depth analysis on chemistry/chemical synthesis, phytochemical/biological activities, and/or biotechnological applications through *in vitro* production of xanthones and their precursors (Mazimba et al., 2013; Li et al., 2017; Gaid et al., 2019; Khattab and Farag, 2020; Tovilovic-Kovacevic et al., 2020; Pinto et al., 2021). Additionally, a review by El-Seedi et al. (2010) covers various biosynthetic pathways in different organisms including plants, but a more updated review is needed considering the current findings in literature within the recent decade. Therefore, this review mainly covers research articles up to 2021, specifically on the topic of xanthone biosynthesis in plants, of which were screened from Web of Science, Scopus, Pubmed and Google Scholar. Furthermore, biosynthetic enzymes related to the xanthone pathway, isolated and/or characterized from xanthone-producing plants are also gathered (Table 1) and discussed to highlight the current advancement, and future direction toward completing the xanthone pathway.

**TABLE 1:** Enzymes involved in the xanthone pathway characterized from various xanthone-producing plants.

Enzymes	Species	Detection level	Citations
Phenylalanine ammonia-lyase (PAL)	*Hypericum androsaemum*	Biochemical	Abd El-Mawla et al., 2001; Abd El-Mawla and Beerhues, 2002
	*Hypericum perforatum, Hypericum canariense*	Biochemical	Klejdus et al., 2013
Cinnamate-CoA ligase (CNL)	*Hypericum androsaemum*	Biochemical	Abd El-Mawla and Beerhues, 2002
	*Hypericum calycinum*	Molecular (heterologous expression in *E. coli*)	Gaid et al., 2012
Cinnamoyl-CoA hydratase/lyase (CHL)	*Hypericum androsaemum*	Biochemical	Abd El-Mawla and Beerhues, 2002
Benzaldehyde dehydrogenase (BD)	*Hypericum androsaemum*	Biochemical	Abd El-Mawla and Beerhues, 2002
	*Hypericum calycinum*	Molecular (heterologous expression in *E. coli*)	Singh et al., 2021
Benzoate-CoA ligase (BZL)	*Hypericum androsaemum*	Biochemical	Abd El-Mawla and Beerhues, 2002
	*Hypericum calycinum*	Molecular (heterologous expression in *E. coli*)	Singh et al., 2020
3-Hydroxybenzoate-CoA ligase (3BZL)	*Centaurium erythraea*	Biochemical	Barillas and Beerhues, 1997, 2000
	*Hypericum androsaemum*	Biochemical	Schmidt and Beerhues, 1997
Benzophenone synthase (BPS)	*Hypericum calycinum*	Biochemical	Klingauf et al., 2005
	*Centaurium erythraea*	Biochemical	Beerhues, 1996
	*Garcinia mangostana*	Molecular (heterologous expression in *E. coli*)	Nualkaew et al., 2012; Songsiriritthigul et al., 2020; Klamrak et al., 2021
	*Hypericum androsaemum*	Molecular (heterologous expression in *E. coli*)	Liu et al., 2003
	*Hypericum perforatum*	Molecular (heterologous expression in *E. coli*)	Tocci et al., 2018
	*Hypericum sampsonii*	Molecular (heterologous expression in *E. coli*)	Huang et al., 2012
Benzophenone 3′-hydroxylase (B3′H)*,**	*Hypericum androsaemum*	Biochemical	Schmidt and Beerhues, 1997
1,3,5-Trihydroxyxanthone synthase (1,3,5-THXS)*	*Centaurium erythraea*	Biochemical	Peters et al., 1997
1,3,7-Trihydroxyxanthone synthase (1,3,7-THXS)**	*Hypericum androsaemum*	Biochemical	Peters et al., 1997
Cytochrome P450 81AA1 (CYP81AA1)**	*Hypericum perforatum, Hypericum calycinum*	Molecular (heterologous expression in *S. cerevisiae*)	El-Awaad et al., 2016
Cytochrome P450 81AA2 (CYP81AA2)*	*Hypericum perforatum*	Molecular (heterologous expression in *S. cerevisiae*)	El-Awaad et al., 2016
Xanthone 6-hydroxylase (X6H)	*Centaurium erythraea*	Biochemical	Schmidt et al., 2000a
Aromatic Prenyltransferase (aPT)	*Hypericum calycinum*	Molecular [heterologous expression in baculovirus-infected insect cells (*S. frugiperda*, *Sf9*)]	Fiesel et al., 2015
8-Prenylxanthone-forming prenyltransferase (PT8PX)	*Hypericum sampsonii, Hypericum calycinum*	Molecular [heterologous expression in *S. cerevisiae* (*H. sampsonii*) and *N. benthamiana* (*H. calycinum*)]	Nagia et al., 2019
Patulone-forming prenyltransferase (PTpat)	*Hypericum sampsonii, Hypericum calycinum*	Molecular (heterologous expression in *S. cerevisiae* (*H. sampsonii*) and *N. benthamiana* (*H. calycinum*)	Nagia et al., 2019
Norathyriol 6-*O*-glucosyltransferase (StrGT9)	*Gentiana triflora*	Molecular (cell-free protein expression system)	Sasaki et al., 2021
Malonyl-CoA acyltransferase (StrAT2)	*Gentiana triflora*	Molecular (cell-free protein expression system)	Sasaki et al., 2021
C-Glycosyltransferase (CGT)	*Mangifera indica*	Molecular (heterologous expression in *E. coli*)	Chen et al., 2015

These enzymes are classified either detected at the biochemical level (enzymatic activities of partially-purified or crude protein extracts from native sources) or detected at the molecular level (coding sequence isolation followed by in vitro protein expression and enzymatic activity assays). Similar enzymes but with different naming are indicated by single or double asterisks (*,**).

## Biosynthesis of Xanthone Core Structures in Plants

**FIGURE 2:**
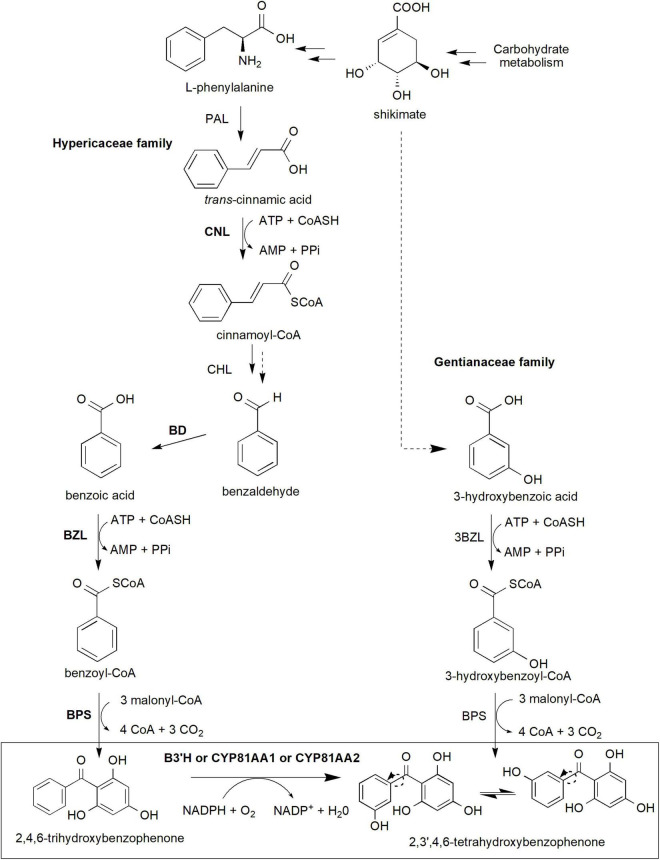
The core xanthone biosynthesis pathway in plants. The shikimate pathway supplies shikimate and L-phenylalanine precursors (detailed pathway is provided in Supplementary Figure 1) to produce benzophenone intermediates, in particular 2,3′,4,6-tetrahydroxybenzophenone isomers used for downstream xanthone biosynthesis. In Gentianaceae family, 3-hydroxybenzoic acid is formed from shikimate and subsequently to 3-hydroxybenzoyl-CoA and later 2,4,5′6-tetrahydroxybenzophenone. Meanwhile, Hypericaceae family utilizes L-phenylalananine-dependent pathway through several more reactions to produce 2,4,6-trihydroxybenzophenone and later the 2,3′,4,6-tetrahydroxybenzophenone. This latter reaction requires B3′H activity of which can be catalyzed by both CYP81AA1 or CYP81AA2 enzymes in Hypericum. Multiple arrows indicate multiple steps between intermediates while dotted arrows indicate hypothesized/proposed pathways. Protein activities that have been detected at molecular level are shown in bold while normal font type indicates protein activities detected at biochemical level (refer to Table 1). The two arrows (one unbroken line and one dotted) for Cinnamoyl-CoA hydratase/lyase (CHL) indicate that the enzymatic reaction has been characterized at the biochemical level from a crude protein extract, but whether this involves one or two enzymatic steps is yet to be validated at the molecular level. 3BZL, 3-hydroxybenzoate-CoA ligase; ATP, adenosine triphosphate; AMP, adenosine monophosphate; BD, benzaldehyde dehydrogenase; BZL, benzoate-CoA ligase; B3′H, benzophenone 3′-hydroxylase; BPS, benzophenone synthase; CHL, cinnamoyl-CoA hydratase/lyase; CNL, cinnamate-CoA ligase; CoASH, coenzyme A; NADPH, reduced nicotinamide adenine dinucleotide phosphate; PAL, phenylalanine ammonia-lyase; PPi, inorganic pyrophosphate.

Xanthone biosynthesis in plants generally occurs *via* the established shikimate pathway, which links carbohydrate metabolism to aromatic compound biosynthesis (Supplementary Figure 1; Kumar et al., 2015; Fu et al., 2017; Li et al., 2017). Precursor compounds from glycolysis (phosphoenolpyruvate) and pentose phosphate pathway (erythrose 4-phosphate) are used in the synthesis of shikimate and subsequently L-phenylalanine through an elaborate pathway involving various enzymes and intermediates (Supplementary Figure 1). These precursors are important to generate benzophenone intermediates especially 2,3′,4,6-tetrahydroxybenzophenone (2,3′,4,6-tetraHBP; Figure 2), a central intermediate for xanthone biosynthesis in plants (El-Seedi et al., 2010). Interestingly, classical studies using radioactively labeled precursor compounds, such as [^14^C]L-phenylalanine, [^14^C]benzoic acid, and [^14^C]hydroxybenzoic acid, among others, showed that the pathway to produce the 2,3′,4,6-tetraHBP can vary between plant species (Atkinson et al., 1968; Gupta and Lewis, 1971; Abd El-Mawla et al., 2001; Abd El-Mawla and Beerhues, 2002). For example, Hypericaceae family (e.g., *Hypericum androsaemum*, *H. calycinum*, and *H. sampsonii*) mainly utilizes benzoic acid from  L-phenylalanine to produce the benzophenone, whereas Gentianaceae family (*Centaurium erythraea* and *Swertia chirata*) uses 3-hydroxybenzoic acid from shikimate as a precursor compound (Figure 2; Abd El-Mawla et al., 2001; Wang et al., 2003; Singh et al., 2020).

In the L-phenylalanine-dependent pathway (Hypericaceae), the amino acid is converted to *trans*-cinnamic acid by the action phenylalanine ammonia-lyase (PAL) enzyme (Abd El-Mawla et al., 2001; Abd El-Mawla and Beerhues, 2002; Figure 2). PAL is the first committed step in the phenylpropanoid pathway, enabling dedicated carbon flux toward specialized metabolism in plants including xanthone biosynthesis (Gaid et al., 2012; Maeda and Dudareva, 2012; Lynch and Dudareva, 2020). However, despite a number of molecular studies (heterologous expression or homologous overexpression) has been conducted in various plant species (Hyun et al., 2011; Castro et al., 2020), PAL detection and characterization have mainly been conducted at the biochemical level in *Hypericum* spp. (Abd El-Mawla et al., 2001; Abd El-Mawla and Beerhues, 2002; Klejdus et al., 2013). Inhibition of the enzyme using 2-aminoindane-2-phosphonic acid in *Hypercium perforatum* and *Hypercium canariense* resulted in the significant reduction of total soluble phenols as well as benzoate and cinnamate derivatives (Klejdus et al., 2013), suggesting the central importance of PAL in the phenylpropanoid metabolism. Subsequently, the synthesis of cinnamoyl-CoA from the *trans*-cinnamic acid will be catalyzed by cinnamate-CoA ligase (CNL; Gaid et al., 2012). The *CNL* coding sequence has been previously cloned and characterized from various species including *H. calycinum* (Gaid et al., 2012), *Arabidopsis thaliana* (Lee et al., 2012), *Petunia hybrida* (Klempien et al., 2012) and *Malus* x *domestica* “Golden delicious” (Teotia et al., 2019).

The cinnamoyl-CoA intermediate is then synthesized to benzoyl-CoA which requires three more enzymatic reaction steps involving cinnamoyl-CoA hydratase/lyase (CHL), benzaldehyde dehydrogenase (BD) and benzoate-CoA ligase (BZL; Abd El-Mawla and Beerhues, 2002; Singh et al., 2020). Although the three proteins have been proven to exist and functioning at the biochemical level from various species (Abd El-Mawla and Beerhues, 2002; Beuerle and Pichersky, 2002; Gaid et al., 2009, 2019; Saini et al., 2017, 2020), only BD and BZL have been verified at the molecular level (coding sequence isolation followed by *in vitro* heterologous expression and enzymatic assays) in *H. calycinum* (Singh et al., 2020, 2021) and in snapdragon (*Antirrhinum majus*; BD only; Long et al., 2009). The latter *BZL* gene, also known as *HcAAE1* (*acyl-activating enzyme 1*), has been shown to increase its expression prior to xanthone accumulation post-elicitation, suggesting its role upstream of the xanthone pathway (Singh et al., 2020). Additionally, the enzyme prefers benzoic acid substrate and is localized subcellularly at both peroxisomes and cytosol indicating the activation of CoA-dependent non-β-oxidative route for the benzoyl-CoA production (Singh et al., 2020).

Subsequent reaction by a Type III polyketide synthase called benzophenone synthase (BPS) condenses the benzoyl-CoA molecule with three malonyl-CoA resulting in the formation of 2,4,6-trihydroxybenzophenone (2,4,6-triHBP or also known as phlorbenzophenone; Figure 2; Beerhues et al., 2007; Beerhues and Liu, 2009; Nualkaew et al., 2012). The BPS enzyme was previously cloned from *H. androsaemum*, *H. perforatum, H. sampsonii*, and *G. mangostana* before its enzymatic activity and/or subcellular localization characterized (Liu et al., 2003; Klingauf et al., 2005; Huang et al., 2012; Nualkaew et al., 2012; Belkheir et al., 2016; Tocci et al., 2018; Klamrak et al., 2021). The expression of *BPS* has been shown to precede the increase in xanthone accumulation and that the protein and xanthone products were majorly localized to the exodermis region of the *H. perforatum* root, suggesting their roles as the first line of defense against soilborne pathogens (Tocci et al., 2018). Recently, the crystal structures of BPS from both *H. androsaemum* and *G. mangostana* were reported, further revealing their function and specificity toward benzoyl-CoA substrate to synthesize 2,4,6-triHBP (Stewart et al., 2017; Songsiriritthigul et al., 2020).

The 2,4,6-triHBP intermediate is then converted to 2,3′,4,6-tetraHBP by a cytochrome P450 (CYP) monooxygenase known as CYP81AA that possesses benzophenone 3′-hydroxylase (B3′H) activity (Figure 2; Schmidt and Beerhues, 1997; El-Awaad et al., 2016). Interestingly, two homologous CYP81AA enzymes exist, CYP81AA1 and CYP81AA2 in *Hypericum* spp. and have a bifunctional role to catalyze another downstream compound, either 1,3,7-trihydroxyxanthone (1,3,7-THX) or 1,3,5-trihydroxyxanthone (1,3,5-THX), respectively (El-Awaad et al., 2016).

Meanwhile, in the Gentianaceae family, the biosynthetic pathway originates from shikimate to produce 3-hydroxybenzoic acid *via* L-phenylalanine-independent pathway as confirmed by radioactively labeled precursors (Abd El-Mawla et al., 2001; Wang et al., 2003; Figure 2). The latter compound is then thioesterified to 3-hydroxybenzoyl-CoA by 3-hydroxybenzoate-CoA ligase (3BZL) in the presence of ATP and CoA, and subsequently, sequential condensation by BPS leads to the formation of 2,3′,4,6-tetraHBP (Barillas and Beerhues, 1997; Wang et al., 2003; Li et al., 2017). However, both 3BZL and BPS remain yet to be investigated at the molecular level in the Gentianaceae family, and only the former enzyme (3BZL) had been biochemically validated in *C. erythraea* (Beerhues, 1996; Barillas and Beerhues, 1997). Furthermore, the 3BZL could only efficiently activate 3-hydroxybenzoic acid rather than benzoic acid as substrates (Barillas and Beerhues, 1997), suggesting the activation of the L-phenylalanine-independent pathway in this *Centaurium* species. On the other hand, *Aquilaria* spp. from the Thymelaeaceae family is predicted to produce core xanthone structure through 4-hydroxybenzoyl-CoA, instead of the 3-hydroxybenzoyl-CoA (Li et al., 2021; Supplementary Figure 2).

The 2,4,6-triHBP and 2,3′4,6-tetraHBP are precursor compounds to various benzophenones (Supplementary Figure 3). These compounds, such as sampsonione A, hypercalin A, 2,4′,4,6-tetraHBP and 2,3′,4,4′,6-pentaHBP (maclurin), garcinol, and guttiferone A, are known to exhibit various biomedical and pharmaceutical benefits, such as antitumor (Rukachaisirikul et al., 2005; Koeberle et al., 2009; Lay et al., 2014; Behera et al., 2016) and anti-inflammatory (Liao et al., 2005; Pardo-Andreu et al., 2011), among others.

**FIGURE 3:**
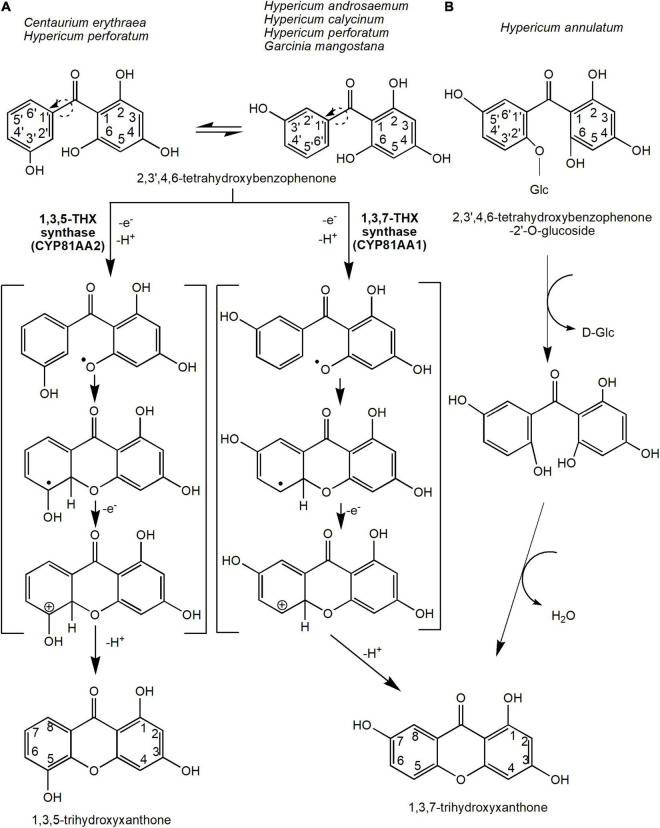
The reaction mechanisms for the two main precursors of xanthones, 1,3,5-trihydroxyxanthone and 1,3,7-trihydroxyxanthone. This oxidative phenol coupling reaction is present in several plant species including *C. erythraea*, *H. androsaemum*, **H. calycinum, H.* perforatum*, and *G. mangostana*. **(A)** In addition, 1,3,7-trihydroxyxanthone can be formed through the deglycosidation process of 2,3′,4,6-tetrahydroxybenzophenone-2′-O-glucoside in *H. annulatum*. **(B)** Dotted arrows indicate hypothetical/proposed pathways while protein activities detected at the molecular level are in bold (refer to Table 1). CYP, cytochrome P450; THX, trihydroxyxanthone.

Oxidative phenol coupling reaction involving 2,3′,4,6-tetraHBP ring closure that occurs either at the *ortho* or *para* position of the 3′-OH group forms the two main core xanthone structures, 1,3,5-THX and 1,3,7-THX, respectively (Figure 3A). In *C. erythraea*, 2,3′,4,6-tetraHBP was shown at the biochemical level to regioselectively cyclize to 1,3,5-THX, whereas in *H. androsaemum*, (Peters et al., 1997; El-Awaad et al., 2016) and *G. mangostana* to 1,3,7-THX (Atkinson et al., 1968; Gupta and Lewis, 1971; Beerhues and Liu, 2009). The reaction mechanism that underlies the regioselective intramolecular mediated oxidative coupling during cyclization of the benzophenone is proposed to involve two stages of one-electron oxidation (Figure 3A). The loss of the first one-electron and a deprotonation produces a phenoxy radical, which cyclizes benzophenone through electrophilic attack (Figure 3A). Then, the hydroxy-cyclohexadienyl radical intermediate loses an electron and a proton to generate the 1,3,5-THX and 1,3,7-THX compounds (Peters et al., 1997; El-Seedi et al., 2010).

The enzymes that catalyze these reactions are originally known as xanthone synthases belonging to the CYP oxidases (Peters et al., 1997). However, they were later renamed to 1,3,5-THX synthase (or CYP81AA2) and 1,3,7-THX synthase (or CYP81AA1), respectively (El-Awaad et al., 2016; Khattab and Farag, 2020). These CYP81AA homologs isolated from *H. perforatum* and *H. calycinum* were expressed in yeast by El-Awaad et al. (2016), who showed that six substrate recognition sites are responsible for the regiospecificity of the enzymatic reactions, especially for the CYP81AA2. Interestingly, both enzymes were found in *Hypericum* spp. transcriptome databases (Gaid et al., 2012; El-Awaad et al., 2016). This suggests that both isomeric products could actually be synthesized in any one species, perhaps dependent upon certain physiological responses or signals. Recently, a metabolomics study in mangosteen also found putative 1,3,5-THX and a derivative of 1,3,7-THX at different tissues and stages of ripening (Mamat et al., 2020), further corroborating that certain species may possess both enzymes for the two different cyclization reactions. In the future, the application of sequencing efforts at either the genomics or transcriptomics levels on these xanthone-producing plants will undeniably help the identification and characterization of these vital biosynthetic enzymes.

Alternatively, 1,3,7-THX is proposed to be generated spontaneously from a different precursor compound such as 2,4,5′,6-tetrahydroxybenzophenone-2′-*O*-glucoside in *Hypericum annulatum* (Kitanov and Nedialkov, 2001; El-Seedi et al., 2010; Figure 3B). The glucoside group at the 2′ position of the 2,4,5′6-tetrahydroxybenzophenone-2′-*O*-glucoside molecule is first removed by enzymatic or acidic hydrolysis before cyclization of both rings (Kitanov and Nedialkov, 2001; Figure 3B).

**FIGURE 4:**
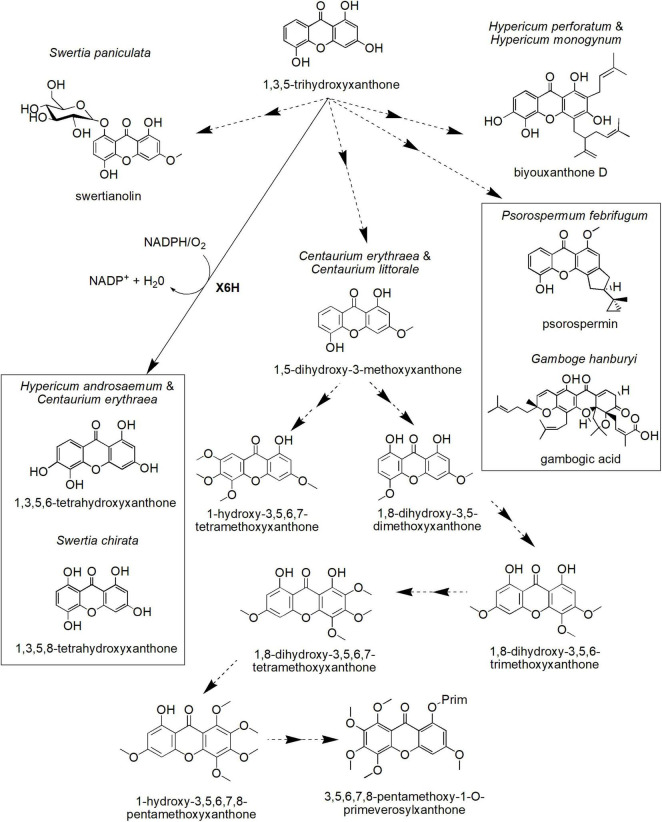
Proposed biosynthetic pathways of several xanthone derivatives derived from 1,3,5-trihydroxyxanthone precursor. X6H, xanthone 6-hydroxylase. Dotted arrows indicate hypothetical/proposed pathways while protein activities detected at the molecular level are in bold (refer to Table 1).

These xanthones (1,3,5-THX and 1,3,7-THX) are the main precursors of most other xanthones; therefore, this intramolecular cyclization contributes an essential branch point from benzophenone intermediate to xanthone biosynthesis (Figure 3A). However, it is to be noted that most of the downstream xanthone pathway from these core xanthone precursors (Figures 3, 4) are only proposed reactions and only a handful of enzymes have been biochemically characterized previously (Table 1).

## Biosynthetic Pathways of Xanthone Derivatives in Plants Through 1,3,5-Trihydroxyxanthone

One of the xanthone core compounds, 1,3,5-THX, can give rise to many more different xanthone structures (Figure 4). For instance, 1,3,5-THX can be hydroxylated to produce 1,3,5,6-tetrahydroxyxanthone in *H. androsaemum* and *C. erythraea* (Schmidt et al., 2000a), a compound known to induce diuresis and saluresis (Mariano et al., 2019, 2021). This process is catalyzed by the plant-specific CYP-dependent monooxygenase known as xanthone 6-hydroxylase (X6H) that has been biochemically determined to require NADPH and O_2_ (Barillas and Beerhues, 2000; Schmidt et al., 2000b). In *S. chirata*, 1,3,5-THX is also hydroxylated but at the C-8 position of the ring, contributing to 1,3,5,8-tetrahydroxyxanthone, the key xanthone in this species (Figure 4; Velíšek et al., 2008). The compound is known to be a potent inhibitor for angiopoietin-like protein 3 pathway to regulate ketosis, a metabolic disorder due to ketone body accumulation (Xiao et al., 2012).

On the other hand, the cell cultures of *C. erythraea* and *Centaurium littorale* treated with methyl jasmonate as well as yeast extract differentially accumulated other types of xanthones, such as 1,5-dihydroxy-3-methoxyxanthone and 1-hydroxy-3,5,6,7-tetramethoxyxanthone (Figure 4; Beerhues and Berger, 1995). Moreover, the cell cultures of *C. erythraea* accumulated 3,5,6,7,8-pentamethoxy-1-*O*-primeverosyl-xanthone (Figure 4), which is in parallel with its cell growth (Beerhues and Berger, 1994). A hypothetical scheme for this compound (3,5,6,7,8-pentamethoxy-1-*O*-primeverosylxanthone) was proposed by Beerhues and Berger (1995). This includes intermediates, such as 1,8-dihydroxy-3,5-dimethoxyxanthone, 1,8-dihydroxy-3,5,6-trimethoxyxanthone, 1,8-dihydroxy-3,5,6,7-tetramethoxyxanthone, and 1-hydroxy-3,5,6,7,8-pentamethoxyxanthone (Figure 4). However, the full list of enzymes involved in this process is still unknown.

In addition to that, 1,3,5-THX is proposed to form more complex xanthones, such as biyouxanthone D, gambogic acid, psorospermin, and swertianolin with diverse biological activities (Figure 4). For instance, biyouxanthone D, a polyprenylated xanthone isolated from *in vitro* root cultures of *H. perforatum* and field-grown roots of *Hypercium monogynum* has been shown to possess antifungal activity (Tocci et al., 2013) and neuroprotective effects (Xu et al., 2016). Another prenylated xanthone, gambogic acid, the main bioactive compound for *Garcinia hanburyi*, has been observed to induce apoptosis in many types of cancer cell lines including BGC-823 human gastric cancer line (Liu et al., 2005), human hematoma SMMC-7721 cells (Guo et al., 2004), and prostate tumor (Yi et al., 2008). Recently, gambogic acid has been shown to exert such cytotoxic mechanism against the cancer cell lines by inducing paraptosis, a cell death induced by vacuolization (Seo et al., 2019). Furthermore, psorospermin (Figure 4) isolated from *Psorospermum febrifugum* also demonstrated a significant antitumor and anti-leukemic activities in mice (Anywar et al., 2021). This woody plant that originated from Africa has been identified to be effective to be used as an anti-pyretic, a leprosy treatment, a poisoning treatment, and a purgative material. Meanwhile, swertianolin (1,5-dihydroxy-3-methoxyxanthone-8-*O*-ββ-D-glucopyranoside), a glycosylated xanthone, is the active compound isolated from felworts (*Swertia paniculata*) (Pant et al., 2011). The plant is known for its use as a bitter tonic in Indian traditional medicine as well as for the treatment of certain mental illnesses including melancholia (Pant et al., 2011). Thus, these xanthone compounds could be developed into potential drugs in treating various ailments in the future, but more investigation toward elucidating the identity and activity of respective biosynthetic enzymes may be conducted to allow sustainable *in vitro* or *in vivo* production of these compounds.

## Biosynthetic Pathways of Xanthone Derivatives in Plants Through 1,3,7-Trihydroxyxanthone

**FIGURE 5:**
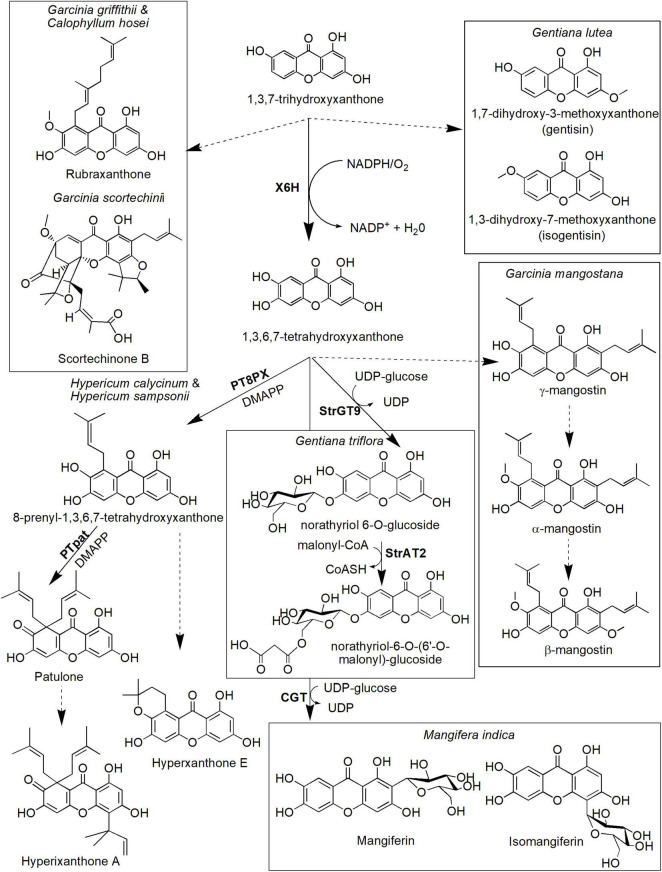
Proposed biosynthetic pathways of several xanthone derivatives derived from 1,3,7-trihydroxyxanthone precursor. Dotted arrows indicate hypothetical/proposed pathways while protein activities detected at the molecular level are in bold (refer to Table 1). CoASH, coenzyme A; DMAPP, dimethylallyl pyrophosphate; PT8PX, 8-prenylxanthone-forming prenyltransferase; PTpat, patulone-forming prenyltransferase; StrAT2, malonyl-CoA acyltransferase; StrGT9, norathyriol 6-O-glucosyltransferase; UDP, uridine diphosphate; X6H, xanthone 6-hydroxylase.

Many more xanthone derivatives are derived from the other core structure, 1,3,7-THX (Figure 5). For instance, 1,3,7-THX is proposed to be a precursor compound for prenylated xanthones, such as rubraxanthone and scortechinone B, as well as simple xanthones, such as 1,7-dihydroxy-3-methoxyxanthone (gentisin) and 1,3-dihydroxy-7-methoxyxanthone (isogentisin; Figure 5). Rubraxanthone is mainly isolated from *Garcinia* (Jantan et al., 2002; Susanti et al., 2014) and *Calophyllum* species (Daud et al., 2021; Zailan et al., 2021) and showed significant ability to inhibit platelet aggregation in human whole blood samples (Alkadi et al., 2013). Meanwhile, scortechinone B extracted from the *Garcinia scortechinii*’s stem, bark, and latex exhibited surprisingly strong antimicrobial activity toward a methicillin-resistant *Staphylococcus aureus* strain with minimum inhibitory concentration (MIC = 2 μg/mL) compared to vancomycin antibiotic (MIC = 3.13–6.25 μg/mL; Rukachaisirikul et al., 2005; Araújo et al., 2019). Furthermore, an experiment toward *Gentiana lutea* rhizome has identified the presence of xanthone compounds called gentisin and isogentisin mainly derived from the 1,3,7-THX (Figure 5; Atkinson et al., 1968; Mudrić et al., 2020). Gentisin exhibited potent inhibition against β-glucuronidase enzyme that plays a critical role in drug metabolism and irinotecan-induced diarrhea (Sun et al., 2020), whereas isogentisin has been shown to protect endothelial injury caused by smoking (Schmieder et al., 2007).

The hydroxylation of 1,3,7-THX also forms 1,3,6,7-tetrahydroxyxanthones in various species including *H. androsaemum* and *G. mangostana* (Figure 5; Schmidt et al., 2000a; El-Awaad et al., 2016). This process is catalyzed by the same X6H enzyme that catalyzes hydroxylation of 1,3,5-THX earlier (Barillas and Beerhues, 2000; Schmidt et al., 2000b) and potentially resided in the endoplasmic reticulum (Schmidt et al., 2000a; El-Awaad et al., 2016). In *G. mangostana*, prenylation of the 1,3,6,7-tetrahydroxyxanthones at the C-2 and C-8 positions is proposed to generate γ-mangostin, and subsequent O-methylation at the hydroxyl group of C-7 produces α-mangostin (Nualkaew et al., 2012; Mazimba et al., 2013), the main xanthone compounds in this species (Mazlan et al., 2019; Mamat et al., 2020). We postulate that another main xanthone in this species called β-mangostin may directly follow the same route of which another O-methylation could occur at the C-3 hydroxyl group position. Whether similar route is present in other β-mangostin-producing species such as *Calophyllum* spp. (Gómez-Verjan et al., 2017; Zailan et al., 2021) still needs further investigation. These xanthones (α-, β- and γ-mangostin) are known to possess antitumor, antioxidant, antidiabetic, antimicrobial, and anti-inflammatory properties, among others (Aizat et al., 2019; Marzaimi and Aizat, 2019). Unfortunately, specific enzymes that catalyzed these prenylation and methylation reactions in mangosteen have yet to be identified, although ongoing transcriptomics and proteomics work in this species (Abdul-Rahman et al., 2017; Jamil et al., 2021) could shed some lights onto answering this question soon.

The other pathway leading from 1,3,6,7-tetrahydroxyxanthones is the biosynthesis of patulone, hyperxanthone E, and hyperixanthone A that are isolated from *Hypericum* spp. (Fiesel et al., 2015; Nagia et al., 2019; Figure 5). Recently, aromatic prenyltransferase (aPT) enzymes from *H. calycinum* and *H. sampsonii* were transformed into *Nicotiana tabacum* and *Saccharomyces cerevisiae* (Nagia et al., 2019). One of the enzymes, 8-prenylxanthone-forming prenyltransferase (PT8PX), was shown to exhibit prenylation activity and mainly localized at the envelope of the chloroplast (Nagia et al., 2019). The reaction product, 8-prenyl-1,3,6,7-tetrahydroxyxanthone, is proposed to be cyclized to become hyperxanthone E or further prenylated by patulone-forming prenyltransferase (PTpat) to patulone (Fiesel et al., 2015; Nagia et al., 2019). Both prenylation reactions by PTpat and earlier PT8PX require dimethylallyl pyrophosphate (DMAPP) as a prenyl donor (Nagia et al., 2019). Interestingly, PTpat has been shown to be able to catalyze patulone directly from 1,3,6,7-tetrahydroxyxanthone *via gem*-diprenylation, but this reaction is not preferred (Nagia et al., 2019). The patulone compound may then be converted to hyperixanthone A by means of reverse prenylation (Nagia et al., 2019), but further characterization of the responsible enzyme is needed. These xanthone derivatives have also shown pharmaceutical potential, for instance, hyperxanthone E has cytotoxic activity against breast cancer and human lung tumor cell lines (Tanaka et al., 2004) as well as a potent anti-inflammatory agent (Zhang et al., 2014). This compound also can be induced by phytopathogens (Janković et al., 2002; Gaid et al., 2012) and accumulated in plant callus and cell suspension upon hormonal induction (Dias et al., 2000). Meanwhile, patulone was able to inhibit platelet-activating factor responsible for asthma and inflammation (Oku et al., 2005; Singh et al., 2013), whereas hyperixanthone A is a potent anti-bacterial agent against *S. aureus* (Xiao et al., 2008; Xin et al., 2011).

Additionally, another prenyltransferase from *Morus alba* called isoliquiritigenin-3′-dimethylallyltransferase (IDT) also has shown regiospecific prenylation of the 1,3,6,7-tetrahydroxyxanthones to generate 2-dimethylallyl-1,3,7-trihydroxyxanthone that can act as a strong neuroprotective agent (Wang et al., 2016). However, *M. alba* is not known physiologically to produce xanthone which suggests substrate promiscuity property of the IDT enzyme (Wang et al., 2016). Other plant PTs have also been characterized from various species including *Cudrania tricuspidata* (Wang et al., 2014), *Artemisia capillaris* (Munakata et al., 2019), *Citrus* x *paradisi* (Munakata et al., 2021), *Humulus lupulus* (Li et al., 2015), and *Cannabis sativa* (Gülck et al., 2020) but their roles have yet to be elucidated for xanthone prenylation.

Meanwhile, two enzymes responsible for the biosynthesis of two xanthone glucosides, norathyriol 6-O-glucoside(also known as tripteroside or Xt1) and norathyriol-6-O-(6′-O-malonyl)-glucoside (called Xt2) were recently characterized at the molecular level by Sasaki et al. (2021). The first enzyme, norathyriol 6-O-glucosyltransferase (StrGT9) mediates the glucosylation of 1,3,6,7-tetrahydroxyxanthone to Xt1 compound. This reaction requires UDP-glucose as a donor molecule for the glucose moiety. The Xt1 compound will then be malonylated in the presence of malonyl-CoA to Xt2 by the second enzyme called malonyl-CoA acyltransferase (StrAT2). Interestingly, the products of these enzymatic reactions (Xt1 and Xt2) contribute to the red coloration in cultivated Japanese gentians (*Gentiana triflora*), together with anthocyanin co-pigmentation (Sasaki et al., 2021). Mangiferin, a well-known C-glucoside xanthone, can also be synthesized from 1,3,6,7-tetrahydroxyxanthones (Figure 5). It is originally isolated from mango *M. indica* L. (Anacardiaceae), and its structure was established as 2-C-β-D-glucopyranosyl-1,3,6,7-tetrahydroxyxanthone after extensive chemical reaction and spectroscopic investigation (Bhatia et al., 1967; Ehianeta et al., 2016). Isomangiferin, its structural isomer isolated from *A. asphodeloides*, was characterized as 4-C-β-D-glucopyranosyl-1,3,6,7-tetrahydroxyxanthone. Mangiferin was among the first xanthone that was discovered to display a wide range of medicinal properties including activation of the central nervous system (Bhattacharya et al., 1972) and antioxidant, antibiotic, anti-inflammatory, antiproliferative, antidiabetic, chemopreventive, analgesic, and immunomodulatory activities (Khare and Shanker, 2016; Saha et al., 2016). The formation of mangiferin in *A. asphodeloides* was investigated by means of feeding experiments, and the biosynthetic route for mangiferin and related xanthone C-glycosides has been studied and proposed by Fujita and Inoue (1980). In their route, maclurin 3-C-glucoside is postulated to be an intermediate and has been enzymatically converted to mangiferin and isomangiferin. More recently, an enzyme called C-glycosyltransferase (CGT) that catalyzes this reaction has been isolated and characterized from *M. indica* (Chen et al., 2015). The enzyme has been shown to exhibit substrate promiscuity to specific benzophenones and xanthones, suggesting its prominent role in catalyzing several biosynthetic reactions within the species (Chen et al., 2015).

## Conclusion

This review details the biosynthetic process of xanthone in plants, which has yet to be updated comprehensively in the last decade. The biosynthesis of these xanthones can be either originated from shikimate precursor (L-phenylalanine-independent pathway) as shown in Gentianaceae family or through L-phenylalanine-dependent pathway as evidenced in Hypericaceae family. The pathway also involved benzophenone intermediates, followed by a regioselective oxidative mediated intramolecular coupling to form the xanthone ring structures, 1,3,5-THX and 1,3,7-THX. Several xanthone derivatives can be originated from these xanthone precursors and may differ between plants. In the future, this resource will allow genetic engineering of xanthone biosynthesis in microbial cell factory, hence providing sustainable option for producing this valuable bioactive compound.

## Author Contributions

JR analyzed, interpreted, and reviewed the research articles as well as drafted the article. IS analyzed and critically reviewed the manuscript drafts. WA designed the research framework and critically revised the manuscript. All authors contributed to the manuscript editing and revision and approved the final version of the manuscript.

## Conflict of Interest

The authors declare that the research was conducted in the absence of any commercial or financial relationships that could be construed as a potential conflict of interest.

## Publisher’s Note

All claims expressed in this article are solely those of the authors and do not necessarily represent those of their affiliated organizations, or those of the publisher, the editors and the reviewers. Any product that may be evaluated in this article, or claim that may be made by its manufacturer, is not guaranteed or endorsed by the publisher.

## Footnotes

1
http://dnp.chemnetbase.com/faces/chemical/ChemicalSearch.xhtml
